# Introducing the FAIR Principles for research software

**DOI:** 10.1038/s41597-022-01710-x

**Published:** 2022-10-14

**Authors:** Michelle Barker, Neil P. Chue Hong, Daniel S. Katz, Anna-Lena Lamprecht, Carlos Martinez-Ortiz, Fotis Psomopoulos, Jennifer Harrow, Leyla Jael Castro, Morane Gruenpeter, Paula Andrea Martinez, Tom Honeyman

**Affiliations:** 1 Research Software Alliance, QLD 4780 Cairns, Australia; 2 Software Sustainability Institute & EPCC, University of Edinburgh, 47 Potterrow, Edinburgh, EH8 9BT UK; 3 NCSA & CS & ECE & iSchool, University of Illinois at Urbana-Champaign, 1205 W Clark St., Urbana, IL 61801 USA; 4 Institute of Computer Science, University of Potsdam, An der Bahn 2, 14476 Potsdam, Germany; 5 Netherlands eScience Center, Science Park 140, 1098 XG Amsterdam, Netherlands; 6 Institute of Applied Biosciences, Centre for Research and Technology Hellas, Thessaloniki, 57001 Greece; 7 ELIXIR Hub, South Building, Wellcome Genome Campus, Hinxton, Cambridgeshire CB10 1SD UK; 8 Semantic Technologies team, ZB MED Information Centre for Life Sciences, Gleueler Strasse 60, 50931 Cologne, Germany; 9 Software Heritage, Inria, 2 rue Simone IFF, Paris, 75012 France; 10 Research Software Alliance/Australian Research Data Commons, Level 6, Duhig Tower, The University of Queensland, Brisbane, QLD 4072 Australia; 11 Australian Research Data Commons, University of Technology Sydney Library, Ultimo, NSW 2007 Australia

**Keywords:** Policy, Research management

## Abstract

Research software is a fundamental and vital part of research, yet significant challenges to discoverability, productivity, quality, reproducibility, and sustainability exist. Improving the practice of scholarship is a common goal of the open science, open source, and FAIR (Findable, Accessible, Interoperable and Reusable) communities and research software is now being understood as a type of digital object to which FAIR should be applied. This emergence reflects a maturation of the research community to better understand the crucial role of FAIR research software in maximising research value. The FAIR for Research Software (FAIR4RS) Working Group has adapted the FAIR Guiding Principles to create the FAIR Principles for Research Software (FAIR4RS Principles). The contents and context of the FAIR4RS Principles are summarised here to provide the basis for discussion of their adoption. Examples of implementation by organisations are provided to share information on how to maximise the value of research outputs, and to encourage others to amplify the importance and impact of this work.

## Introduction

In 2016 the publication of “The FAIR Guiding Principles for scientific data management and stewardship”^1^ supported a vision where valuable scientific outputs are made ‘FAIR’ by becoming more Findable, Accessible, Interoperable and Reusable. From the outset, the FAIR Guiding Principles were intended to be applicable to many kinds of digital assets. Increased understanding of the importance of research software in research has catalysed application of the FAIR Guiding Principles to this type of digital asset.

Community-endorsed FAIR principles for research software were released in 2022 by the FAIR for Research Software (FAIR4RS) Working Group (WG), which was jointly convened by the Research Software Alliance (ReSA), Future Of Research Communications and E-Scholarship (FORCE11), and the Research Data Alliance (RDA). This milestone reflects the maturation of the research community in understanding the benefits of having FAIR research software, and coming together as the FAIR4RS WG to achieve this. The FAIR4RS WG is a global and interdisciplinary community whose members share an interest in the application of FAIR principles to research software, such as researchers, software users, developers and maintainers, policy makers, infrastructure support staff, and funders.

The FAIR4RS Principles are relevant to any stakeholder in the research community seeking to increase transparency, reproducibility, and reusability of research. This paper highlights the importance of the FAIR4RS Principles and the positive signals of adoption that demonstrate high levels of community support. It must also be acknowledged that research software and data discoverability is a long-standing challenge and there have been multiple efforts in the past to address it^2,3^. In this sense, the FAIR4RS principles provide an umbrella framework that integrates aspects of these existing efforts.

The paper is organised as follows. The Results section briefly presents the FAIR4RS Principles, and provides examples of how they can be applied to different types of research software. The Discussion section considers their importance and impact, and provides organisational examples of implementation in practice that readers can learn from and utilise. Finally, the Methods section discusses how the process used to develop the FAIR4RS Principles both leveraged and amplified community understanding of the critical role of research software in maximising research value, leading to the very positive signals of early adoption.

## Results

In this section, each of the FAIR4RS Principles is proposed and explained, as contained in version 1.0^4^. First, each foundational principle (F, A, I and R) is described, followed by the numbered guiding principles used to detail it. It should be noted that the FAIR4RS Principles are aspirational, as are the FAIR Guiding Principles. The community has come together to provide clarity around common goals, by defining simple and research software appropriate goalposts to inform those who publish and/or preserve research software. Contextual information follows, then examples of how the principles can be applied to three types of research software are presented.

Research software is defined by the FAIR4RS WG as including “source code files, algorithms, scripts, computational workflows and executables that were created during the research process or for a research purpose. Software components (e.g., operating systems, libraries, dependencies, packages, scripts, etc.) that are used for research but were not created during or with a clear research intent should be considered software in research and not Research Software. This differentiation may vary between disciplines”^5^.

### FAIR4RS Principles

The FAIR4RS Principles^2^ are:

**Table:** 

**F: Software, and its associated metadata, is easy for both humans and machines to find**.
F1. Software is assigned a globally unique and persistent identifier.
F1.1. Components of the software representing levels of granularity are assigned distinct identifiers.
F1.2. Different versions of the software are assigned distinct identifiers.
F2. Software is described with rich metadata.
F3. Metadata clearly and explicitly include the identifier of the software they describe.
F4. Metadata are FAIR, searchable and indexable.
**A: Software, and its metadata, is retrievable via standardised protocols**.
A1. Software is retrievable by its identifier using a standardised communications protocol.
A1.1. The protocol is open, free, and universally implementable.
A1.2. The protocol allows for an authentication and authorization procedure, where necessary.
A2. Metadata are accessible, even when the software is no longer available.
**I: Software interoperates with other software by exchanging data and/or metadata, and/or through interaction via application programming interfaces (APIs), described through standards**.
I1. Software reads, writes and exchanges data in a way that meets domain-relevant community standards.
I2. Software includes qualified references to other objects.
**R: Software is both usable (can be executed) and reusable (can be understood, modified, built upon, or incorporated into other software)**.
R1. Software is described with a plurality of accurate and relevant attributes.
R1.1. Software is given a clear and accessible license.
R1.2. Software is associated with detailed provenance.
R2. Software includes qualified references to other software.
R3. Software meets domain-relevant community standards.

### FAIR research software examples

Three examples of how research software projects implement the principles are provided to increase understanding of how they apply in practice. It should be noted that the application of the FAIR4RS Principles is the responsibility of the owners (who are often the creators) of the software, not the users. However, scholarly infrastructures are needed to provide certain functionalities to apply FAIR to software. The principles can be applied to a wide range of research software, and the examples here are of a command-line tool for a specific task, a collection of scripts and notebooks that form a complex research software product, and graphical user interfaces to other packages or libraries. The following three examples show how parts of the FAIR4RS Principles can be implemented.

- Comet is a command-line tool and desktop application for tandem mass spectrometry sequence database search^6^. It is registered in the bio.tools catalogue of bioinformatics tools, where it has a globally unique and persistent identifier (FAIR4RS Principle: F1), and rich metadata (F2) that includes the identifier (F3) and is searchable and indexable (F4). Comet can be downloaded via the browser following the links provided in the metadata using https (A1). The metadata in bio.tools is independent from the Comet repository, and will stay accessible should the software itself become inaccessible (A2). Comet uses standard data types from the proteomics domain for its input and output data (I1) that are documented in the metadata as functional annotations (I2). The software is licensed under the Apache 2.0 open source licence, and the publicly accessible project repository on GitHub includes detailed information about its development (R1). The code includes dependencies to external software packages, such as Thermo Scientific’s MSFileReader library (R2).
- PuReGoMe is a project aimed at understanding Dutch public sentiment during the COVID-19 outbreak period by analysing real-time Twitter data^7^. It provides a collection of Python scripts and Jupyter notebooks for this purpose. PuReGoMe has a (versioned) DOI from Zenodo (F1) and is registered in the Research Software Directory that captures the most relevant metadata (F2), including the identifier (F3), in searchable and indexable form (F4). The software can be downloaded from the project repository (A1), while metadata is accessible independently from the registry (A2). PuReGoMe uses standard file formats (e.g., CSV files) for data exchange (I1) and refers to other objects such as websites (I2). The project uses the Apache 2.0 open source licence, and the GitHub repository has detailed records of the development history (R1). The code includes dependencies to other software, such as various Python libraries (R2).
- gammaShiny is an application that provides enhanced graphical user interfaces for the R gamma package^8^. It is used to process *in-situ* gamma-ray spectrometry measurements for luminescence dating. gammaShiny has been deposited in the HAL French national archive and it has a persistent globally unique identifier^8^ (F1) with the HAL identifier of the metadata record and a SWHID, identifying specifically the software artefact on the Software Heritage universal software source code archive. Thanks to the HAL platform, where a licence is mandatory, gammaShiny is under a GNU General Public Licence v3.0 (R1). The archived versions of gammaShiny’s source code in Software Heritage include a codemeta.json file, identifiable with a SWHID, where other metadata is available including dependencies named in CodeMeta - ‘softwareRequirements’(R2).

## Discussion

This section discusses the importance and impact of the FAIR4RS Principles in reflecting research community maturation, and provides adoption examples for readers to learn from to facilitate easier implementation.

### Research community maturation

The development of the FAIR4RS Principles is a milestone for the research community in recognizing the increasing value of research software as fundamental and vital to research worldwide. While improving the practice of scholarship is a common goal of the open science, scientific software, and FAIR communities, and while making research software more FAIR can improve research, research software is only now emerging as a strong focus of FAIRness.

While many of the FAIR Guiding Principles could be directly applied to research software by treating software and data as similar digital research objects, unique characteristics of software (e.g., its executability, composite nature, continuous evolution and versioning), as well as of the ecosystem in which it is developed and shared (e.g., social coding platforms, package management systems), made it necessary to revise and extend the principles to create the FAIR4RS Principles. For example, while the process of making data FAIR is typically done when the data is published to an archive, open source software ideally should start working toward satisfying the FAIR4RS Principles when it is initially being developed since it may be used by others directly from its development environment.

An additional benefit of the development of community-endorsed principles is catalysing shared practices. Enabling community participation increases awareness of the challenges faced by different stakeholders and how the principles might address them. The FAIR4RS WG identified a range of opportunities for future work^9,10^ that share an emphasis on the need for increased standardisation of community practices. These areas include metadata and identifier authority, metadata vocabularies and metadata properties, software identifiers, domain-relevant community standards for software and identification targets. This discussion will make it simpler for people wanting to follow the principles to know how to do so.

### Adoption

The FAIR4RS WG has been successful in facilitating initial adoption of its outcomes, reflecting high levels of initial impact. Adoption and implementation of the FAIR4RS Principles will create significant outcomes for many stakeholders, ranging from increased clarity for funders around their own requirements for software investments to guidelines for publishers and research institutions on sharing requirements. The FAIR4RS Principles are also relevant to the larger ecosystem and the stakeholders that support research software (e.g., repositories and registries). A number of groups and organisations are planning to adopt the FAIR4RS Principles, including ELIXIR, Australian Research Data Commons (ARDC), Netherlands eScience Center (NLeSC), and ZB MED, and are at various stages at this point, as described in this subsection. This demonstrates the initial organisation support that the FAIR4RS Principles are receiving, and provides information on policies, guidelines and activities that other organisations could consider implementing.

The FAIR4RS Working Group completed its work with the release of the community validated principles in May 2022. The RDA Software Source Code Interest Group is the maintenance home for the FAIR4RS Principles. Concerns or queries about the principles can be raised at RDA plenary events organised by this interest group, where there may be opportunities for adopters to report back on progress. In two years time, the community will gather again to see if anything needs to be changed.

#### Australian research data commons

The ARDC is a national facility enabled by the Australian Government’s National Collaborative Research Infrastructure Strategy. The mission of the ARDC is to accelerate research and innovation by driving excellence in the creation, analysis and retention of high-quality datasets. To support this, the research software program at the ARDC is working towards recognition of research software as a first-class output of research. The ARDC National Agenda for Research Software includes activities seeking to make research software outputs more FAIR in Australia^11^. The ARDC is undertaking a number of actions to implement the FAIR4RS Principles, beginning with policy change. ARDC’s co-investment policy lays out expectations for partners to make the outputs of projects receiving ARDC co-investment more FAIR. ARDC’s policy for FAIR outputs is being updated to reference the FAIR4RS Principles as appropriate for software outputs arising from future co-investment.

In terms of implementation, the ARDC provides national support for adopting the FAIR Guiding Principles through collaborations, activities and guidance materials. This support is expanding to include the FAIR4RS Principles as well as development of an equivalent guidance for Virtual Research Environments (VREs) through the RDA FAIR4VREs WG. The initial focus for engagement is through the ARDC platforms community, which represents 26 projects developing national platforms infrastructure with a total investment of AUD$58M. This will be followed with activities targeting broader research software communities across Australia. Activity around materials development has already begun. A FAIR research software counterpart is being developed to complement the highly successful ARDC FAIR data self-assessment tool. This new tool will be used to raise awareness of the FAIR4RS Principles, and to help a wide variety of stakeholders identify and order actions to target in adopting the FAIR4RS Principles. Finally, while not currently a major producer of research software itself, the ARDC will make some of its own software outputs FAIR to demonstrate the effort and impact of doing so.

#### ELIXIR

ELIXIR coordinates and develops life science resources across Europe so that researchers can more easily find, analyse and share data, exchange expertise, and implement best practices. As a European intergovernmental organisation that is made up of life scientists, computer scientists, and support staff, ELIXIR unites Europe’s leading life science organisations to help researchers take advantage of the huge amounts of data produced in life science, to gain new insights into how living organisms work in health and disease. This is achieved by coordinating, integrating and sustaining bioinformatics resources across ELIXIR member states and enabling users in academia and industry to access services that are vital for their research.

Advancing the understanding of life and disease over all the domains in life sciences requires that research data, analysis tools, standards and computational services adhere to the FAIR Guiding Principles. ELIXIR policy, whilst having data as the focal point, explicitly notes that “most of the principles outlined are equally applicable to other research assets such as software, training materials and other digital research objects”^12^, and recommends that all research outputs of ELIXIR infrastructure be FAIR, including a strong recommendation to all ELIXIR partners that software developed and supported by ELIXIR be FAIR. This includes standalone tools (such as InterMine), platforms (such as Galaxy) as well as services (such as bio.tools and OpenEBench). Moreover, and in the context of the FAIR assessment and adoption process, of particular interest are services that enable and support FAIRness in research outcomes, such as FAIRsharing. This is an RDA-endorsed resource, recommended by ELIXIR and other organisations across all disciplines. In the context of research software, FAIRsharing covers: (i) the standards used to describe the software metadata; (ii) the standards used as software input and output formats; and (ii) the software code repositories.

Additionally, one of the most recent achievements of the ELIXIR Tools Platform is the ELIXIR Software Management Plan^13^, a low-barrier standard specifically tailored for life science researchers for capturing the life cycle for the software that is produced within the lifetime of a particular project or activity^13^. As a clear outcome of ELIXIR adopting the FAIR4RS Principles, a roadmap has been put in place to ensure that the ELIXIR Software Management Plan, currently implemented as a standalone tool based on the Data Stewardship Wizard^14^, is fully aligned to them. Specifically, the sections and individual questions of the Software Management Plan will be clearly annotated to the corresponding FAIR Principles, in order to both increase the awareness of the principles themselves, as well as promote FAIRness for software within ELIXIR.

There are also several training efforts under the ELIXIR Training Platform related to FAIR; FAIR Data Management and Data Stewardship training, FAIR Training, the Terms4FAIRskills initiative, etc. A new set of training materials will be developed within the next two years to increase the use of Software Management Plans and understanding of the underlying FAIR4RS principles.

#### Netherlands eScience Center

The Netherlands eScience Center is the Dutch national centre of research software expertise, and has an internal strategy for Open Science and Software Sustainability. This document highlights the centrality of software quality, reusability, and adoptability to the eScience Center’s vision. The FAIR4RS Principles are a key element of this vision particularly related to software reusability.

The eScience Center is adopting the FAIR4RS Principles by:

- Using the FAIR4RS Principles to support the creation of reusable software as part of its calls and projects. All projects funded by the eScience Center are required to have Software Management Plans.
- Contributing to developing the skills necessary to implement the FAIR4RS Principles through skills and knowledge development activities: digital skills training, development of practical guidelines, etc. Their Digital Skills Programme helps develop some of the necessary skills required to develop software that is as reusable as possible.
- Updating the Five Recommendations for FAIR software tool^15^ to better align with the FAIR4RS Principles.
- Promoting the principles on relevant policy agendas at a (inter)national level. For example, the eScience Center is collaborating with the Dutch Research Council (NWO) in the creation of national templates for Software Management Plans. The aim of these templates is to provide guidance for Dutch research organisations and scientific communities, as well as individual researchers, on how to organise research software and ensure its sustainability. The FAIR4RS Principles are being considered as a starting point in the development of these templates. The guidance provided by the templates will be, wherever possible, closely aligned with the FAIR4RS Principles.

#### ZB MED

ZB MED Information Centre for Life Sciences is a German national infrastructure and research centre for information and data in the life sciences. The mission of ZB MED is to ensure the national provision of information and literature in the fields of medicine, health care, nutritional, environmental and agricultural sciences – including the relevant basic sciences and related subject areas – for the purposes of research, teaching and practical application. To support this, ZB MED follows and encourages an open science policy so that all research outcomes become open to the public, and adhere to the FAIR Guiding Principles and good research management practices. However, data is only one piece of the puzzle. For research to become reproducible and sustainable, connected FAIRification is needed across all the pieces, including data, software, workflows and other research objects.

ZB MED promotes the FAIR Guiding Principles by providing support, advice and training to researchers in this regard. ZB MED is now adopting and promoting the FAIR4RS Principles to better support research software. Researchers at ZB MED already follow some practices related to FAIR and openness, including the recommendations for open source code^16^, combined with the Five Recommendation for FAIR Software tool^15^. Currently, ZB MED is adapting and extending these practices to better align them to the FAIR4RS Principles and the adoption guidelines that the same group is working on. Furthermore, ZB MED plans to extend dissemination activities around FAIR to include the FAIR4S Principles so that more researchers, at national and international level, understand and adopt them.

## Methods

The adoption of the FAIR4RS Principles by a range of organisations reflects the extensive consultation undertaken by the FAIR4RS WG with the research community. The FAIR4RS WG has engaged about 500 people (from more than 110 organisations over more than 34 countries) in the development of the principles, including the more than 240 FAIR4RS WG members. “The FAIR4RS team: Working together to make research software FAIR”^17^ and the FAIR4RS Community Profile^18^ provide details on the approach to community collaboration, showcasing a model for teamwork across the research software community.

The FAIR4RS WG efforts began with subgroups from July 2020 to March 2021 that provided outputs to support the development of the FAIR4RS Principles. This work was brought together in a single report and presented for feedback by the wider FAIR4RS community in March 2021^10^. Draft FAIR4RS Principles were released in June 2021^19^ and underwent a formal community review process for one month. The current version of the FAIR4RS Principles, as summarised here, is a cumulative result of this work.

Part of the reason for the FAIR4RS WG’s very high levels of success in community engagement is because it brought together a range of efforts to apply aspects of FAIR to research software since 2017^10^, and because it sought to align with a range of FAIR data efforts. In this way the FAIR4RS WG was able to leverage and amplify existing community momentum to demonstrate the benefits of implementing the principles.

## Data Availability

No data is shared in this paper.

## Code availability

No code is shared in this paper.
